# Research on interaction of innovation spillovers in the AI, Fin-Tech, and IoT industries: considering structural changes accelerated by COVID-19

**DOI:** 10.1186/s40854-022-00403-z

**Published:** 2023-01-05

**Authors:** Chi-Ming Ho

**Affiliations:** Department of Finance, Southern Taiwan University of Science and Technology, Tainan, Taiwan

**Keywords:** AI, Covid-19, Fin-Tech, Innovation spillover, IoT, G12, O14, O23, O33

## Abstract

This paper aims to probe the influence of innovation spillovers in the artificial intelligence (AI) and financial technology (Fin-tech) industries on the value of the internet of things (IoT) companies. Python was utilized to download public information from Yahoo Finance, and then the GARCH model was used to extract the fluctuations of cross-industry innovation spillovers. Next, the Fama–French three-factor model was used to explore the interactive changes between variables. The panel data regression analysis indicates that the more firms accept innovation spillovers from other industries, the better the excess return; however, this effect differs because of industrial attributes and the environmental changes induced by COVID-19. Additionally, this study finds that investing in large-cap growth stocks of IoT firms is more likely to yield excess returns. Finally, the study yields lessons for policy leverage to accelerate the upgrading and transformation of innovation-interactive industries by referring to the practices of Singapore and South Korea.

## Introduction

The process of industrial development can be situational and is a common phenomenon (Bloom et al. 2013), which Arrow (1972) refers to as an innovation spillover. The channels for innovation spillover may be geographical convenience, cross-shareholding, conference participation, learning, or research and development (R&D) collaboration (Cohen and Levinthal 1989; Matray 2021; Shiraishi and Yano 2021). When firms receive the innovation achievements of others through such channels, they can increase their market share and benefit from cost reductions (Jaffe 1986; Bernstein and Nadiri 1989); however, investor pricing errors, innovation patterns, policy uncertainty, and public R&D expenditures affect the relationship between innovation spillovers and stock returns (Hirshleifer and Jiang 2010; Diebold and Yilmaz 2012; Stambaugh and Yuan 2017; Chen et al. 2020; Rehman and Narayan 2021). Nevertheless, the concept of how to transmit across industries and set model factors desperately requires clarification in literature and deserves further research.

Many studies have indicated that volatility indices (VIX) can transmit the latest developments in a specific market, as well as the spillover of such information (Crawford and Sobel 1982), to change the prices of other marketable securities indirectly (Campbell and Vuolteenaho 2004; Lin 2021; Massa and Zhang 2021; Blomstrom and Persson 1983; Globerman 1979; Hong et al. 2007). Industry indices have received particular attention from studies covering specific industries’ innovation information. The innovation spillover process deserves further attention (Blomstrom and Persson 1983; Globerman 1979; Hong et al. 2007). Diebold and Yilmaz (2012) explored cross-industry innovation spillovers using the forecast error variance decomposition of a vector autoregression (VAR) model; however, their results could not highlight the pure innovation spillover effects on the industry’s stock return because they did not exclude the model’s common factors. In addition, their conclusion and the VAR model assumption do not clarify the dependent variables in the model; however, according to Chan et al. (1990), Chen et al. (2013) and Jiang et al. (2015), it is clear that the spillover effect is the independent variable, and the abnormal payoff can be the dependent variable. Considering the relationship between variables and excluding the common explanatory factors, the capital asset pricing model (CAPM) (Sharpe 1964; Lintner 1965; Black 1972; Fama and French 1996) is introduced as a feasible framework for analysis.

The COVID-19 pandemic has ravaged the global financial market and changed economic structures (Dai et al. 2021; Ramelli and Wagner 2020; Almeida 2021). Firms use internet of things (IoT) technologies to enhance productivity and reduce costs to survive such transformation (Fleisch 2010); IoT is a system of interconnected devices, machinery, and digital machines that are already collecting various kinds of data. Artificial Intelligence (AI) uses existing data to analyze trends. The underlying technology of fin-tech firms is the blockchain, which can store data to prevent tampering (Ho 2020). Theoretically, if a firm can combine these three aspects to perfectly realize data collection, analysis, storage, and information security and intends to drive business transformation (Fleisch 2010; Jiao et al. 2021), it can undoubtedly become the most competitive firm (Ferreira et al. 2021; Schumpeter 2000). For the above reasons, this study selects an appropriate model to link cross-industry innovation spillover effects and environment change factors, explains the less covered areas, and fills some research gaps in the extant literature.

This paper has three contributions. The previous literature mentioned that enterprises accept the methods of innovation spillovers for common reasons, such as a favorable geographic position, technological cooperation, participation in meetings, employment of key personnel, foreign shareholding, or industry clustering, which are all major corporate decisions. The fluctuations and backward effect of digital measurements might result in inadequate short-term benefits and abnormal long-term returns. This study reveals that individual stocks extracted with cross-industry indices can replace companies’ composite pipelines to accept innovation spillovers and interpret the abnormalities in asset pricing (Hirshleifer and Jiang 2010). Moreover, when the environment changes, the model can be used to observe different enterprise adjustments in the acceptance of innovation spillovers, which past studies have not mentioned.

Furthermore, while CAPM has been using firm characteristics to explain stock return, it is limited to firms’ financial ratios (Daniel et al. 1997). This study demonstrates that, in the case of environmental changes, some companies’ acceptance of the degree of innovation fluctuations and responses to internal transformations or organizational reforms change, and such variations are important factors affecting companies’ value. It is possible to link CAPM to the digital economy with factors not yet present in the famous factor zoo’s 150 factors, as proposed by Feng et al. (2020). Finally, collecting real-time or cross-industry panel data has remained an essential aspect of academic research; however, collecting is becoming increasingly difficult as different databases and data frequencies are involved. This paper used the Python program to collect panel data to solve the problems of cross-industry transmission and the variables’ data frequency. Python boasts good computing efficiency and reduced costs; thus, the program is recommended for the academic community (Ong et al. 2013).

This study is divided into five parts. The first part is the introduction; the second part is the theory and hypotheses; the third part is the description of the research models; the fourth part is the panel data regression that explores the industrial attributes and analyzes the changes in economic structure caused by COVID-19, and the last part includes this study’s conclusions and recommendations for future research.

## Literature review and research hypotheses

IoT is an infrastructure that connects physical objects, such as radio frequency identification, sensors, actuators, and other smart devices, to a network to conduct economic activities (Bhayo et al. 2020), and digital machines capable of implementing digital life through network transmission without human–computer interaction. Such machines are often used in intelligent logistics, manufacturing monitoring, medical diagnostics, intelligent sensors, personal smart bracelets, and social consumption (Mohammad et al. 2021). In addition, the Institute of Electrical and Electronics Engineers (IEEE) defines IoT as “the processing of information through transmission, driving the physical exchange of information with the physical world”. Currently, the literature on IoT focuses on system security, with possible reasons related to the variability between technology and data. As a result, systemic risks cannot be included in the existing risk management and control (Kandasamy et al. 2020). According to the International Data Corporation, the rapid growth of the IoT industry has pushed the value of related IoT firms to its peak, particularly as COVID-19 affects our daily life. Besides the three factors of Fama–French (1996), we examine what other unique characteristics IoT application firms have that differentiate them from others and lead to excess profit. Let us start with the early innovation diffusion theory (Rogers 1995).

According to the theory, the diffusion of innovation occurs in a social system, and the innovation passes on through different communication channels; over time, the people involved in the process share new things to reach a certain level of consensus, leading to innovation (Rogers 1995, 2002). According to Dietzenbacher (2000), innovation spillovers might come from cross-industry diffusion and can affect the labor input of the beneficiary industries or firms, changing their economic performance. Per Matray (2021), innovation in one firm promotes innovation in neighboring firms, and the effect of such innovation spillovers decreases rapidly with increasing distance. Furthermore, changes in employer shareholding and venture capital investments that gradually diffuse knowledge to new firms influence innovation learning. Shiraishi and Yano (2021) examined the capital stock of R&D and the number of R&D personnel in Chinese firms and found that these firms benefit from the spillover effects of foreign firms and the effects of other domestic firms. They indicated that the innovation spillovers of state-owned firms benefit from the innovation input and output of other domestic firms, while innovation spillovers of private firms are more related to foreign firms. Overall, certain channels might influence some firms, which might cause them to be affected as well. Spillover effects are the potential indirect economic benefits related to R&D, employee training, technology transfer, and, more importantly, inter-industry spillovers (Globerman 1979; Blomstrom and Persson 1983). The financial literature bridges this interaction to demonstrate how inter-industry innovation spillovers are transmitted.

Many studies have pointed to spillover effects in financial markets, where information from a particular market is transmitted to another through indices and this generates changes in the commodity prices in other markets. Campbell and Vuolteenaho (2004) found that stock prices are expected to increase when cash flows increase, but high discount rates cause stock prices to fall; moreover, cash flow information in the bond market would spill over to the stock market if the bond market experiences a shock from forecasts of future cash flows. Hong et al. (2007) proposed the gradual-information-diffusion hypothesis to explain industry indices that lead to market indices. According to their findings, investors in the market are not entirely rational; they cannot have all the information and can only focus on specific markets for trading. Nevertheless, a leader–laggard relationship arises because of market efficiency. Hong et al. (2007) support the findings of Eleswarapu and Tiwari (1996). Li et al. (2020) stated that risk spillover effects exist between fin-tech firms and traditional financial institutions during technological progress. They used the Granger causality test in quantiles to examine risk spillovers between the two, using three types of spillover networks. The results indicated that risk spillover from fin-tech firms to financial institutions positively correlates with systematic risks. These results have important policy implications, emphasizing that the supervision of fin-tech firms is essential for maintaining financial stability. According to Zhang and Ding (2021), the linkages among financial commodity prices vary depending on data frequency, and the price trends in different commodity markets are significantly and positively correlated. Such linkages or spillovers are driven by cross-sectional liquidity. Lin (2021) analyzed inter-market contagion and found spillover effects among the Shanghai Stock market and inter-market indices after adopting the MIDAS-GARCH model. The commodity and global shipping markets significantly transmit volatility to the Shanghai Stock Exchange Composite (SSEC) before and after a crash crisis. The domestic currency market’s volatility is significantly contagious to SSEC only after the crash. Massa and Zhang (2021) found spillover effects of Hurricane Katrina’s liquidation bonds on other corporate bonds and the changes in the relative availability of bond and bank financing. Qarni and Gulzar (2021) found asymmetry in the volatility spillover between Bitcoin markets and foreign exchange pairs denominated in six major trading currencies (USD, EUR, JPY, GBP, and AUD). They argued that the alternative currency, Bitcoin, could replace the euro and provide higher portfolio diversification benefits. Conversely, Rehman and Narayan (2021) argued that financial markets and economic policies interact, providing evidence regarding the interrelationships of international oil prices with economic policy uncertainty, consumer sentiments, and U.S. investor sentiment indices.

In terms of integrating innovation diffusion theory with evidence of spillover effects, this study argues that the information about a specific industry will be transmitted as news to another commodity or financial market and change its value through the volatility index. We next examine what innovation spillovers in the AI industry affect IoT firms and make them change themselves to generate profits. An empirical study by Naveed et al. (2017) on U.S. music streaming firms found that the advancement of AI, machine learning, fin-tech, virtual reality, big data, and social media significantly changes the live-music-streaming firms’ ecosystem and the interests of other industries. As per Gupta et al. (2021), IoT can connect a great deal of communication and central data information; however, the sheer volume and complexity of the data concerning each other make it difficult to make timely decisions. They used smartphones to design an AI program with optimized generic algorithms to replace expensive monitoring devices and achieve economic advantages and effectiveness. De Prisco et al. (2021) used the example of a gym to build an intelligent ecosystem integrating IoT and AI (named Gym Intelligence) that provides music to reduce physical exertion during training. They found that physical work is more enjoyable with certain types of music—the more enjoyable the environment for trainers, the lower their physical exertion. Qi et al. (2021) focused on the IoT-integrated medical instruments and found wireless networks rendering Medical Cyber-Physical Systems (MCPS) vulnerable to external attacks and possibly compromising patient privacy in the process. Therefore, AI-assisted identity verification is designed and used in MCPS, which solves security and privacy issues and reduces costs, thus tackling two issues simultaneously. Spanaki et al. (2021) used the associated IoT and AI data management applications to explore the application of Agriculture 4.0 systems in data sharing. They found that this management approach or sharing mechanism could assist managers in promoting strategic transformations. The above evidence shows that combining IoT firms and AI can create better management effectiveness, increase consumer satisfaction, and enable the evolution of the artificial internet of things, thereby opening up a new intelligent business model to create value.

Besides the widespread application of AI in IoT, fin-tech formed using blockchains is another rising star in the digital economy; it affects the operation of financial systems (Ho, 2020) and drives the transformation of firms (Jiao et al. 2021). For example, diverse payment methods, policy sales, production histories, and lending practices are conducted through IoT interactions (Bareisis 2017). Stored-value cards, initially used in convenience stores or transportation, have been transformed into convenient third-party payments for small daily expenditures to reduce the need for holding currency. Moreover, insurance firms have created a new business model by collecting specific customer data using IoT to establish differential-rate policies (Jiao et al. 2021). Life insurance firms use wearable devices to obtain individuals’ behavior patterns, making information of both parties more transparent, reducing the information asymmetry of both parties, building consensus to protect rights, and reducing the costs of supervision and agency. Additionally, bankers also use sensory technologies in IoT to monitor the operation of the value chain of lending firms and implement intelligent supervision, from raw materials to finished products, to reduce the overall information asymmetry. Furthermore, IoT firms raise funds through fin-tech platforms to build network trust and explore business opportunities (Li et al. 2020). Recently, online lending has emerged, where potential borrowers can fill out and upload specific data on a web-based platform; financing firms can then collect non-quantitative information and check it through IoT. For example, many universities issue blockchain graduation certificates that can be used to check the authenticity of the borrowers’ information. In other words, the integration of IoT and fin-tech can enable the stakeholders of a business to share in economic growth and create wealth (Huckle et al. 2016; Sun et al. 2016). Nakashima (2018), who held that integrating fin-tech into IoT applications holds the potential to create new business models and services, supported this view. Marsal-Llacuna (2018) demonstrated the advantages of using blockchain in urban areas. Through policy planning, it can even replace the existing network system. Combined with city-level IoT, blockchain technology is poised to become an essential network in cities. Hughes et al. (2019) suggested that blockchain technology could be extended to other business applications to promote innovation and improve efficiency in preexisting fields. Chen et al. (2019) used patent application data from 2003 to 2017 to classify innovation types by the technologies underlying machine-learning applications, finding that fin-tech can bring considerable application value to innovators. Integrating IoT, Robo-Advisor, and blockchain is the most valuable innovation type. Lim et al. (2021) investigated the relationship between IoT and fin-tech. According to their analysis of consumers’ growth in Apple Pay and Samsung Pay, innovative payment has become the most critical fin-tech application. Additionally, their study found consumers’ perceived safety, knowledge of services, validation, perceived usefulness, and satisfaction to be correlated. In other words, knowledge of fin-tech services and mobile payment security significantly influence consumers’ validation and perceived usefulness.

In conclusion, IoT firms can perform better (Shiraishi and Yano 2021) if they develop different strategies to respond to market changes (Fleisch 2010) based on their interests and the innovation spillovers in the AI and fin-tech industries (Globerman 1979; Blomstrom and Persson 1983; Dietzenbacher 2000; Lu et al. 2021). All else equal, IoT firms more receptive to the innovation spillovers from the AI industry have a head start in developing strategies, data control, automation, drone delivery, and cost control (Fleisch 2010; Qi et al. 2021; Spanaki et al. 2021), which can help increase their future value (FV). In other words, the more exposed an IoT firm is to innovation spillovers from the AI industry, the better the IoT firm’s performance. Furthermore, IoT firms can combine fin-tech technologies to create new business models where buyers and sellers form a consensus to increase consumer satisfaction, improve efficiency, and change FV (Huckle et al. 2016; Hughes et al. 2019; Li et al. 2020; Lim et al. 2021; Sun et al. 2016). All other things being equal, consumers favor IoT firms that can switch between different operating platforms and have the flexibility of smart contracts, digital wallet transfers, peer-to-peer (P2P) loans, e-insurance, or accepting different payment methods, which will increase revenues (Chen et al. 2019). Conversely, fin-tech dependence and information asymmetry between buyers and sellers are negatively correlated. Thus, we can reduce agency and supervision costs to improve corporate profit and FV. In other words, the more an IoT firm receives innovation spillovers from the fin-tech industry, the better its performance. This study proposes Hypothesis (H) 1 to confirm the above statement.

### H1

The more an IoT firm receives innovation spillovers from the AI and fin-tech industries, the better its performance.

In the wake of the COVID-19 pandemic, residents rely heavily on various operating platforms to purchase goods, switch between different methods to place orders, and even use virtual currency for transactions. Moreover, buyers need to check the progress of orders, and sellers need to check production histories for manufacturing planning, all related to fin-tech (Li et al. 2020). Additionally, because of the COVID-19 outbreak, governments have restricted people’s movement, creating a shortage of workers; this also drives industrial IoT, eager to automate or use AI to improve operational difficulties. According to the 2021 Artificial Intelligence Index Report issued by Stanford University, machine-learning technology was used extensively during the COVID-19 pandemic, and the use of AI for hiring and private investment was not negatively affected by the COVID-19 outbreak. Nevertheless, the innovation spillover from the AI and fin-tech industries to service and non-service industries differed somewhat. The reliance on delivery during the pandemic resulted in an explosion of business opportunities for service IoT firms. They were driven to change their business models instantaneously (Jiao et al. 2021), and they preferred short-term plans to solve dilemmas or increase consumer satisfaction in their management decisions (Naveed et al. 2017; De Prisco et al. 2021). Thus, their willingness to accept AI innovations was stronger than before the outbreak. Conversely, fin-tech technologies, such as digital wallets and multi-payment gateways, had already been adopted by many service firms before the COVID-19 outbreak, and the acceptance levels of the innovation spillovers from the fin-tech industry were reduced because of limited resources, time constraints, and problem-solving considerations. Therefore, this study establishes H2 to test the aforementioned statement; however, the opposite is true for the fin-tech industry. Conversely, IoT firms of a non-service (manufacturing) nature focus on improving productivity; thus, AI automation and fin-tech investments require long-term planning (Guo 2018). Additionally, innovation spillovers to non-service IoT firms also rely on regional clusters or significant R&D investments (Aysun and Yom 2021), which is more of a capital investment (long-term) decision (Bareisis 2017; Qi et al. 2021). Therefore, firms’ willingness to accept AI and fin-tech industries increased gradually with the onset of the pandemic rather than changing instantaneously. Therefore, this study establishes H3.

### H2

After the COVID-19 outbreak, service IoT firms have become more receptive to the innovation spillovers from the AI industry; however, their willingness to accept innovation spillovers from the fin-tech industry is gradually decreasing.

### H3

After the COVID-19 outbreak, non-service IoT firms have become more receptive to the innovation spillovers from the AI and fin-tech industries.

## Research model

This section describes the research object, period, variables, and model construction. This study aims to understand whether IoT firms are influenced by the innovation spillovers from industries and the three factors of Fama–French (1996), which are part of the CAPM-featured model series (Daniel et al. 1997). Additionally, this study considers that changes in economic and social patterns (Dai et al. 2021) during the COVID-19 pandemic might also lead to structural changes in industries and stock prices. Through this study, the information content of a specific industry’s development can be explained as having different effects on the firms or the commodity prices of another industry (Dietzenbacher 2000; Qarni and Gulzar 2021; Shiraishi and Yano 2021).

### Research object and period

The object of this study is based on the IoT concept stocks listed on the New York Stock Exchange (NYSE) and the National Association of Securities Dealers Automated Quotations (NASDAQ) in the U.S. The sample firms were selected based on Kevin Ashton’s 1998 definition of IoT. We searched on the webpage of Yahoo Finance and used two key instructions (Internet content and information and information technology services) to screen and deduct the duplicate firms, obtaining 50 firms that met the definition. Then, we ran the Python program to download the firms’ stock prices and financial statements from Yahoo Finance. The financial statements, financial ratios, market capitalization, and stock liquidity data provided by Yahoo Finance were sourced from Morningstar; the historical data and daily updates of U.S. stocks and global indices were sourced from Commodity Systems, Inc. Therefore, this study’s data quality is robust. The variables collected include control variables, such as stock price, number of shares outstanding, risk-free interest rate, book value, total assets, and market value. Moreover, because this study aims to understand the influence of the development of the AI and fin-tech industries on IoT firms, we separately collected the indices of the AI and fin-tech industries to calculate the impact of innovation on specific firms. The AI index is sourced from the AI index 15 compiled by Stanford’s Institute for Human-Centered Artificial Intelligence, and the KBW NASDAQ Financial Technology Index (KFTX) is sourced from the indices compiled by Keefe Bruyette and Woods (KBW). The indices were compiled at different times, and the data frequency of listed firms is inconsistent; therefore, estimation bias is likely to occur if too much data is missing when applying the GARCH model or panel data regression. To avoid these drawbacks, we set the study period from January 2017 to June 2021, covering the pandemic period of COVID-19, and collected 58,638 time-series data.

### Research variables

This research investigates the influences of the AI and fin-tech industries on IoT firms’ stock prices. The model constructed is similar to Ho (2020) but with different core independent variables. In the model, the excess return of IoT firms is the dependent variable; firm size, BMR, and excess return of market are the control variables; and the innovation spillovers received by each firm extracted from the GARCH model are the independent variable. Furthermore, considering the role of COVID-19 and industry category, two dummy variables are added to manipulate the model. The following section illustrates the relevant calculations.

### Calculation of variables

The excess return of IoT firms (${R}_{i}-{R}_{f}$) is obtained by subtracting the risk-free rate $({R}_{f}$) from the daily stock return and is used as the model’s dependent variable. Firm size (SIZE) is defined as the natural logarithm of the market value of IoT firms and is used as the first control variable of the model. BMR helps determine whether a firm issues growth stocks or value stocks. In this study, BMR is the natural logarithm of a firm’s book value divided by its market value; it is used as the second control variable of the model. The excess return of the market (${R}_{m}-{R}_{f}$) is calculated by subtracting the risk-free rate $({R}_{f}$) of the corresponding IoT firm from the expected market return; it is used as the third control variable of the model. The details above are the basic calculations of the three factors of Fama–French (1996). The World Health Organization (WHO) announced the name for the pandemic disease, COVID-19, on February 11, 2019; according to the Wikipedia definition, COVID-19 is a contagious disease caused by severe acute respiratory syndrome coronavirus 2 (SARS-CoV-2). The disease has spread worldwide, leading to an ongoing pandemic. There were early rumors about an unknown coronavirus, but it was not confirmed; therefore, this study uses the time defined by the WHO as the cut-off point. The dummy variable, D, for each firm’s data before February 11, 2019, is defined as 0 and 1 after COVID-19 was confirmed. The reason for not using the number of deaths or infections as a proxy for COVID-19 is that population and level of medical care vary according to epidemic prevention in each region or country. The problem of policy backwardness and the significant fluctuation of the numbers arise when using the aforementioned measurement indicators. Additionally, the probability distribution may be fat-tailed, unfavorable to model estimation. Therefore, this paper adopts the period identification criterion to stabilize the time series. Finally, the dummy variable, H, for industries is differentiated into service and non-service industries based on the Standard Industrial Classification codes for IoT firms; H is defined as 1 if it belongs to the service industry and 0 if it does not. The AI and fin-tech index returns are calculated similarly to the aforementioned IoT stock returns (Ho 2020). The variable values are then substituted into the following formulas (3), (4), and (5) (Bollerslev 1986; Bollerslev et al. 1992) to further analyze the volatility of spillover effects of the two industry indices on IoT firms (Dietzenbacher 2000). In addition, the calculation of related variables is also sorted, as shown in Table 1.

**Table 1:** Data sources for the main research variables

Name of firms (by NASDAQ symbol)	Industry classification	Variables	Data source
ALRM (ALRM), Alphabet (GOOGL), Alteryx (AYX), Facebook (FB), NetEase (NTES), Baidu (BIDU), Match Group. (MTCH), Zillow Group, Inc. (Z), Yandex N.V. (YNDX), iQIYI, Inc. (IQ), IAC/ InterActiveCorp (IAC), Weibo Corporation (WB), Cardlytics, Inc. (CDLX), SINA Corporation (SINA), TechTarget, Inc. (TTGT), EverQuote, Inc. (EVER), Groupon, Inc. (GRPN), Trivago N.V. (TRVG), Qutoutiao Inc. (QTT), Uxin Limited (UXIN), TrueCar, Inc.(TRUE), Liberty TripAdvisor Holdings, Inc.(LTRPA), Thryv Holdings, Inc. (THRY), Liberty TripAdvisor Holdings, Inc. (LTRPB), Gaia, Inc. (GAIA), Perion Network Ltd. (PERI), Points International Ltd. (PCOM), 36Kr Holdings Inc. (KRKR), Luokung Technology Corp. (LKC), Lizhi Inc. (LIZI), IZEA Worldwide, Inc. (IZEA), AutoWeb, Inc. (AUTO), Creatd Inc. (CRTD), Moxian, Inc. (MOXC), Cisco (CSCO), Netflix (NFLX), Fastly (FSLY), and JOYY Inc. (YY)	Service	Excess return (${R}_{i,t}-{R}_{f}$), and ${R}_{i,t}=((\frac{{P}_{i,t}}{{P}_{i,t-1}})-1\times 100\%)$, firm size ${(SIZE}_{i,t})=\mathrm{ln}{\left(Market Value\right)}_{i,t}$, book-to-market ratio (${BER}_{i,t}$) = $\mathrm{ln}(\frac{{BE}_{i,t}}{{ME}_{i,t}})$, excess market return (${R}_{m,t}-{R}_{f}$), and ${R}_{m,t}=((\frac{{P}_{m,t}}{{P}_{m,t-1}})-1\times 100\%)$ asset, AI index return $({R}_{AI,t})=((\frac{{P}_{AI,t}}{{P}_{AI,t-1}})-1\times 100\%)$, and fin-tech index return(${R}_{Fin-Tech,t})=((\frac{{P}_{Fin-Tech,t}}{{P}_{Fin-Tech,t-1}})-1\times 100\%)$ Dummy variable D for the time data of each firm is defined as 0 and as 1 after COVID-19 was confirmed Dummy variable H for industries is defined as 1 if it belongs to the service industry and 0 if it does not ${\sigma }_{i,AI,t}^{2}$, innovation spillover effects from the AI industry ${\sigma }_{i,Fin-Tech,t}^{2}$, innovation spillover effects from the fin-tech industry	*Yahoo finance* https://finance.yahoo.com/ *Python Code* %%capture !pip install yfinance import yfinance as yf df = yf.download("Companys", start = "2017–01-01") df["Close"].plot.line(figsize = (18, 6), grid = True) return_rates = df.pct_change() * 100 df = return_rates return_rates.plot.line(grid = True, figsize = (20, 10)) import pandas as pd df.pct_change().corr() *WHO* https://www.who.int *SIC codes* for IoT firms https://www.osha.gov/data/sic-search Using *MLE* to estimate all the parameters in the conditional variance equation ${\sigma }_{i,t}^{2}$=${\alpha }_{0}+{\alpha }_{1}{\varepsilon }_{i,t-1}^{2}+{\alpha }_{2}{\varepsilon }_{AI,t-1}^{2}+{\beta }_{1}{\sigma }_{i,t-1}^{2}+{\beta }_{2}{\sigma }_{AI,t-1}^{2} ({\sigma }_{i,Fin-Tech,t-1}^{2})$ and extracting the coefficients of the volatility of spillover effects
Jiayin Group Inc. (JFIN), Remark Holdings, Inc. (MARK), Nvidia (NVDA), NXP Semiconductors (NXPI), Intel (INIC), Orion Energy Systems (OESX), Amazon (AMZN), Tesla (TSLA), Impinj (PI), Roku (ROKU), Qualcomm (QCOM), and Honeywell (HON)	Non-service		

The measurement of innovation spillovers is one of the focuses of this study. The spillover effect in Dietzenbacher (2000)’s study was measured by the change of the coefficients in the Input–Output (IO) table—that is, the change in the covariance matrix among industries. This study adopts a similar perspective and considers innovation spillovers at different times. These spillovers transmit information to another market through the volatility of industry indices and generate changes in the FV in that market (Campbell and Vuolteenaho 2004; Hong et al. 2007; Eom et al. 2019; Li et al. 2020; Lin 2021; Massa and Zhang 2021; Qarni and Gulzar 2021). In the literature, the volatility of spillover effects is mostly estimated using the GARCH model, which is applicable given heteroskedasticity demand in the time series and considering cross-sectional data. In this study, panel data can be used for model configuration. Therefore, the common GARCH (1, 1) model is used for estimation, for its extensibility and parsimony, and for its sufficient capability to capture the volatility (Bollerslev 1986; Bollerslev et al. 1992). Based on the above discussion, this study’s mean equation of the GARCH model is designed as a basic market model with its own lagged stock return as the explanatory variable (Hong et al. 2007); all parameters are estimated using maximum likelihood estimation (Bollerslev 1986).

1 $$R_{i,t} = \theta_{0} + \theta_{1} R_{m,t} + \theta_{2} R_{i,t - 1} + \varepsilon_{i,t}$$

is the mean equation describing that stock return is affected by its own lagged stock return and the broad market index return.

2 $$\sigma_{i,t}^{2} = \omega_{0} + \omega_{1} \varepsilon_{i,t - 1}^{2} + \omega_{2} \sigma_{i,t - 1}^{2}$$

is the conditional variance equation, describing that the volatility of stock return is affected by the previous period conditional residual (${\varepsilon }_{t-1}^{2}$) and the previous period conditional variance (${\sigma }_{i,t-1}^{2}$), where ${\theta }_{0}, {\theta }_{1}, {{\theta }_{2},,\omega }_{0},{\omega }_{1}, {\omega }_{2}$ are all equation coefficients. Similarly, the AI index is selected to formulate the mean equation

3 $$R_{AI,t} = \gamma_{0} + \gamma_{1} R_{m,t} + \gamma_{2} R_{AI,t - 1} + \varepsilon_{AI,t}$$

and the conditional variance equation

4 $${ }\sigma_{AI,t}^{2} = \varphi_{0} + \varphi_{1} \varepsilon_{AI,t - 1}^{2} + \varphi_{2} \sigma_{AI,t - 1}^{2}$$

where $\gamma_{0,} \gamma_{1} ,\gamma_{2,} \varphi_{0,} \varphi_{1} ,\varphi_{2}$ are also the coefficients of the equations. After that, the two variables in the conditional variance equation for the AI industry ($\varepsilon_{AI,t - 1}^{2}$ and $\sigma_{AI,t - 1}^{2}$) are placed into the conditional variance Eq. (2) for the stock return to form a new stock variance equation, as follows:

5 $${ }\sigma_{i,t}^{2} = \alpha_{0} + \alpha_{1} \varepsilon_{i,t - 1}^{2} + \alpha_{2} \varepsilon_{AI,t - 1}^{2} + \beta_{1} \sigma_{i,t - 1}^{2} + \beta_{2} \sigma_{AI,t - 1}^{2}$$

where ${\alpha }_{0},{\alpha }_{1},{\alpha }_{2},{\beta }_{1},{\beta }_{2}$ are the coefficients of the new equation; the coefficient ${\beta }_{2}$ is the volatility of spillover effects from the AI industry on each stock. We run a regression to extract the coefficients of volatility spillovers used to express the interactions over time. ${\sigma }_{i,AI,t}^{2}$ is used to denote the innovation spillover effects from the AI industry on stock *i* at time *t*. Similarly, changing the AI index into the fin-tech index and performing the calculation of Eqs. (3), (4), and (5) provide the volatility of spillover effects from the fin-tech industry on each firm. ${\sigma }_{i,Fin-Tech,t}^{2}$ is used to measure the innovation spillover effects from the fin-tech industry on firm *i* in period *t*. Table 1 presents this section’s relevant descriptions, variables, and data sources.

### Research model

This study is based on the three factors of Fama–French (1996), with innovation spillovers from AI and fin-tech industries on a specific firm in period *t − 1*. Additionally, the two core variables are conditional variance or volatility, which the extant literature has used for estimation by adding regression equations (Bali and Engle 2010) and analyzed using the panel data model. We choose period random effects for model selection, considering that serially correlated residuals can make the regression results more reliable. The Hausman test is also required, and the original model is shown in Eq. (6):

6 $$\begin{aligned} R_{i,t} - R_{f,t} \,=\, & \alpha_{0} + \beta_{1} \left( {R_{m,t} - R_{f,t} } \right) + \beta_{2} \ln \left( {SIZE_{i,t} } \right) + \beta_{3} \left( {BMR_{i,t} } \right) \\ + \beta_{4} \left( {\sigma_{i,AI,t - 1}^{2} } \right) + \beta_{5} \left( {\sigma_{i,Fin - Tech,t - 1}^{2} } \right) + D_{i,t} + H_{i,t}^{j} + \varepsilon_{i,t} \\ \end{aligned}$$

where ${R}_{i,t}$ is the stock return of IoT firm *i* in period *t*, and ${R}_{f,t}$ is the risk-free rate in period *t*. ${R}_{m,t}$ is the stock return of market in period *t*, $n{(SIZE)}_{i,t}$ is the market value of IoT firm *i* in period *t*, $n{(BMR)}_{i,t}$ is the BMR of IoT firm *i* in period *t*, and ${\sigma }_{i,AI,t-1}^{2}$ is the innovation spillover effect from the AI industry on IoT firm *i* in period *t − 1*. Furthermore, ${\sigma }_{i,Fin-Tech,t-1}^{2}$ is the innovation spillover effect from the fin-tech industry on IoT firm *i* in period *t − 1*, ${\varepsilon }_{t}$ is the residual of the model, and ${\beta }_{i}$ is the regression coefficient. Three hypotheses are established to examine the results of this study. Among them, H1 intends to prove that the spillover effects from the AI and fin-tech industries affect IoT firms’ stock return, that is, ${H}_{1}$:${\beta }_{4}\ge 0$; ${\beta }_{5}\ge 0$. Next, the data is divided into service and non-service firms according to the dummy variable H for industries. Then, the dummy variable ${D}_{i,t}=D$ is used to observe the difference in spillover effects from AI and fin-tech industries on IoT firms before and after the COVID-19 outbreak. As the panel data is either before the COVID-19 outbreak (D = 0) or after (D = 1), we consolidate the equation into Eqs. (7) and (8) to compare the regression coefficients. Equation (7) for the two periods can be combined into one equation. The data belongs to either before or after the COVID-19 outbreak, D_*pre-covid*_ + D_*covid*_ = 1; Eq. (8) shows that the combined equation can be expanded. It is then divided into service- and non-service-industry featured models for analysis. The approach is similar to that of Ho (2020). ${\beta }_{is(ns)\_covid}$ represents the model coefficient of the service (non-service) industry after the COVID-19 outbreak, ${\beta }_{is(ns)\_pre-covid}$ represents the model coefficient of the service (non-service) industry before the COVID-19 outbreak, and Eq. (1) describes the other variables. Thus, H2 in the service industry (${H}_{2}$:${\beta }_{4s\_covid}-{\beta }_{4s\_pre-covid}\ge 0$; ${\beta }_{5s\_covid}-{\beta }_{5s\_pre-covid}\le 0$) and H3 in the non-service industry (${H}_{3}$:${\beta }_{4ns\_covid}-{\beta }_{4ns\_pre-covid}\ge 0$; ${\beta }_{5ns\_covid}-{\beta }_{5n{s}_{pre}-covid}\ge 0)$ can be verified. In combination,

7 $$\begin{aligned} R_{i,t} - R_{f,t} \,=\, & D_{{pre - {\text{cov}} id}} \left[ \begin{gathered} \alpha_{{0\_pre - {\text{cov}} id}} + \beta_{{1\_pre - {\text{cov}} id}} \left( {R_{m,t} - R_{f,t} } \right) + \beta_{{2\_pre - {\text{cov}} id}} \ln \left( {SIZE_{i,t} } \right) + \beta_{{3\_pre - {\text{cov}} id}} \left( {BMR_{i,t} } \right) \hfill \\ + \beta_{{4\_pre - {\text{cov}} id}} \left( {\sigma_{i,AI,t - 1}^{2} } \right) + \beta_{{5\_pre - {\text{cov}} id}} \left( {\sigma_{i,Fin - Tech,t - 1}^{2} } \right) + \varepsilon_{i,t} \hfill \\ \end{gathered} \right] \\ + D_{{{\text{cov}} id}} \left[ \begin{gathered} \alpha_{{0\_{\text{cov}} id}} + \beta_{{1\_{\text{cov}} id}} \left( {R_{m,t} - R_{f,t} } \right) + \beta_{{2\_{\text{cov}} id}} \ln \left( {SIZE_{i,t} } \right) + \beta_{{3\_{\text{cov}} id}}\left( {BMR_{i,t} } \right) \hfill \\ + \beta_{{4\_{\text{cov}} id}} \left( {\sigma_{i,AI,t - 1}^{2} } \right) + \beta_{{5\_{\text{cov}} id}} \left( {\sigma_{i,Fin - Tech,t - 1}^{2} } \right) + \varepsilon_{i,t} \hfill \\ \end{gathered} \right] \\ \end{aligned}$$

8 $${R}_{i,t}-{R}_{f,t}\,=\,{{\alpha }_{0s(ns)\_pre-covid}+(\alpha }_{0s(ns)\_covid}-{\alpha }_{0s(ns)\_pre-covid})D+{\beta }_{1s(ns)\_pre-covid}{(R}_{m,t}-{R}_{f,t})+({\beta }_{1s(ns)\_covid}-{\beta }_{1s(ns)\_pre-covid})D{(R}_{m,t}-{R}_{f,t})+{\beta }_{2s(ns)\_pre-covid}ln{\left(SIZE\right)}_{i,t}+({\beta }_{2s(ns)\_covidS}-{\beta }_{2s(ns)\_pre-covid})Dln{\left(SIZE\right)}_{i,t}+{\beta }_{3s(ns)\_pre-covid}{(BMR)}_{i,t}+({\beta }_{3s(ns)\_covid}-{\beta }_{3s(ns)\_pre-covid}){D(BMR)}_{i,t}+{\beta }_{4s(ns)\_pre-covid}{({\sigma }_{i,AI}^{2})}_{t-1}+({\beta }_{4s(ns)\_covid}-{\beta }_{4s(ns)\_pre-covid})D{({\sigma }_{i,AI}^{2})}_{t-1}+{\beta }_{5s(ns)\_pre-covid}{({\sigma }_{i,Fin-Tech}^{2})}_{t-1}+({\beta }_{5s(ns)\_covid}-{\beta }_{5s(ns)\_pre-covid})D{({\sigma }_{i,Fin-Tech}^{2})}_{t-1}+{\varepsilon }_{i,t}$$

## Results and discussion

In this section, after loading the data into the model, we subject the research variables to basic statistical analysis, correlation analysis, panel data regression, and robustness analysis. The results are described as follows.

### Basic descriptive statistics

There are seven main variables in this study, namely, the excess return of each stock (${R}_{i}-{R}_{f}$), excess market return (${R}_{m}-{R}_{f}$), firm size (SIZE), BMR, innovation spillovers from the AI industry (${\sigma }_{i,AI}^{2}$), innovation spillovers from the fin-tech industry (${\sigma }_{i,Fin-Tech}^{2}$), and total assets (ASSET). Table 2 summarizes the mean; standard deviation (SD); median, maximum, and minimum values; kurtosis; and skewness of all variables obtained after statistical operations.

**Table 2:** Descriptive statistics of the main research variables

	*(*${R}_{i}-{R}_{f}$*)*	*(*${R}_{m}-{R}_{f}$*)*	*BMR*	*SIZE*	${\sigma }_{i,AI}^{2}$	${\sigma }_{i,Fin-Tech}^{2}$	*ASSET*
Mean	− 0.199499	0. 2443	1.607596	6714.399	3,989,182	6,592,577	19.98299
Median	− 0.020600	− 0.0205	0.436205	7.806300	0.073790	0.310049	8.520000
Maximum	1.708000	0.0935	74.62687	5,699,900	4.84E + 10	1.12E + 11	11,460.00
Minimum	− 26.01780	− 0.1232	0.001992	− 5.260959	− 4.42E + 09	− 1.46E + 10	1.000000
SD	1.876233	2.3628	3.602267	85,296.03	3.91E + 08	7.88E + 08	369.1891
Skewness	− 10.46922	2.28	6.620854	33.45088	103.0933	123.0914	30.95241
Kurtosis	112.9281	12.13	70.92549	1591.144	11,166.87	16,147.59	959.1279
Observations	58,638	1,131	58,638	58,638	58,638	58,638	58,638

The basic statistics of the seven research variables are collated in this table, and the data of mean, SD, median, max., min., kurtosis, and skewness are obtained through software calculations

Table 2 primarily describes the research variables’ descriptive statistics. During the sample period, 58,638 observations on panel data were downloaded from 50 IoT firms; however, the corresponding observations on index data were only 1,131. From the data in Table 2, the mean value of the excess return $$({R}_{i}-{R}_{f})$$ of the IoT firms is about − 0.1994, and the difference between the maximum and the minimum values is significant. Furthermore, the excess return does not follow a normal distribution, and the data are right-skewed, indicating that several observations have large values, which explains why the mean value is larger than the median value. The reason is that the U.S. stock market does not have a price limit; however, it has circuit breakers to halt trading in market indices. Regarding the excess return of index ($${R}_{m}-{R}_{f}$$), we take the NASDAQ market index as an example; the mean value of the excess return *(*$${R}_{m}-{R}_{f})$$ is 0.2443, and the maximum and minimum values are quite different. The reason is that perhaps the pandemic’s vicissitudes make the stocks rise or fall, with more extremes occurring on both tails. Moreover, the data are also right-skewed, similar to the excess stock return. Concerning BMR, the mean value is 1.607596, indicating that American IoT firms issued more value stocks during the sample period; however, firm types vary significantly, as evidenced by an SD of 3.602267. The minimum value is as low as 0.001992, likely Tesla, a firm engaged in the Internet of vehicles with a high market value but low book value. The difference in firm size is even more significant; the data do not follow a normal distribution and are skewed to the right. Concerning the innovation spillover effects from the AI industry ($${\sigma }_{i,AI}^{2}$$), the mean value is 3,989,182, indicating that the AI industry has a high innovation spillover effect on IoT firms; however, the median falls at 0.07379, and the data are right-skewed, indicating that some firms receive a high degree of innovation spillover from the AI industry. Last, regarding the innovation spillovers from the fin-tech industry ($${\sigma }_{i,Fin-Tech}^{2}$$), the mean is 6,592,577, indicating that the fin-tech industry has high innovation spillover effects on IoT firms; the data are even more right-skewed, showing that certain firms are significantly affected by innovation spillovers from the fin-tech industry. Through the subsequent panel data model, this study will determine if industries or the pandemic are responsible for the right skewness of the main research variables.

**Fig. 1:**
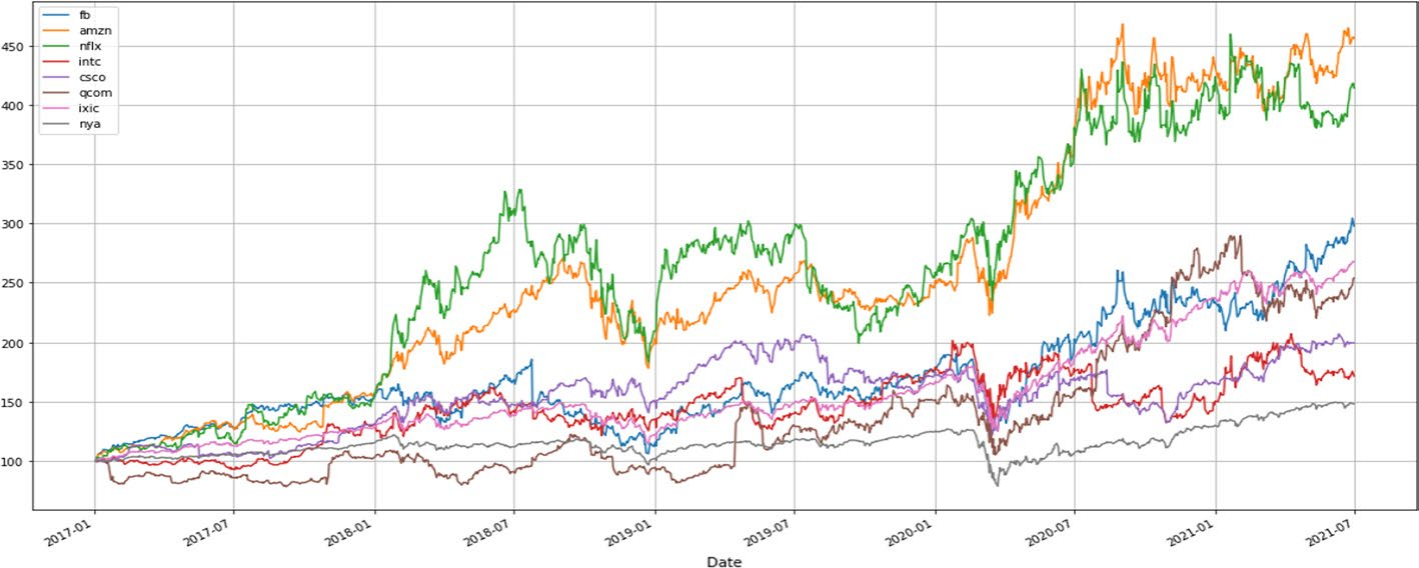
Trend of sample firms relative to stock market index (rebase adjustment)

**Fig. 2:**
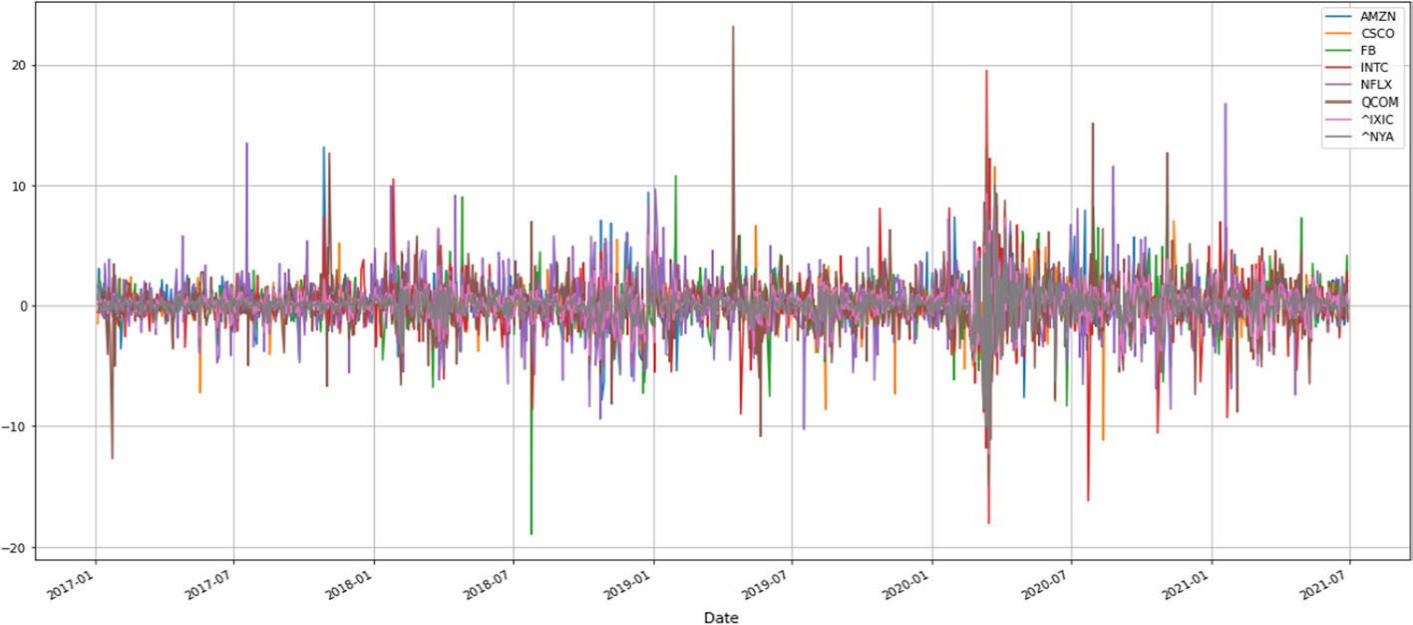
Volatility clustering of sample firms relative to stock market index

**Fig. 3:**
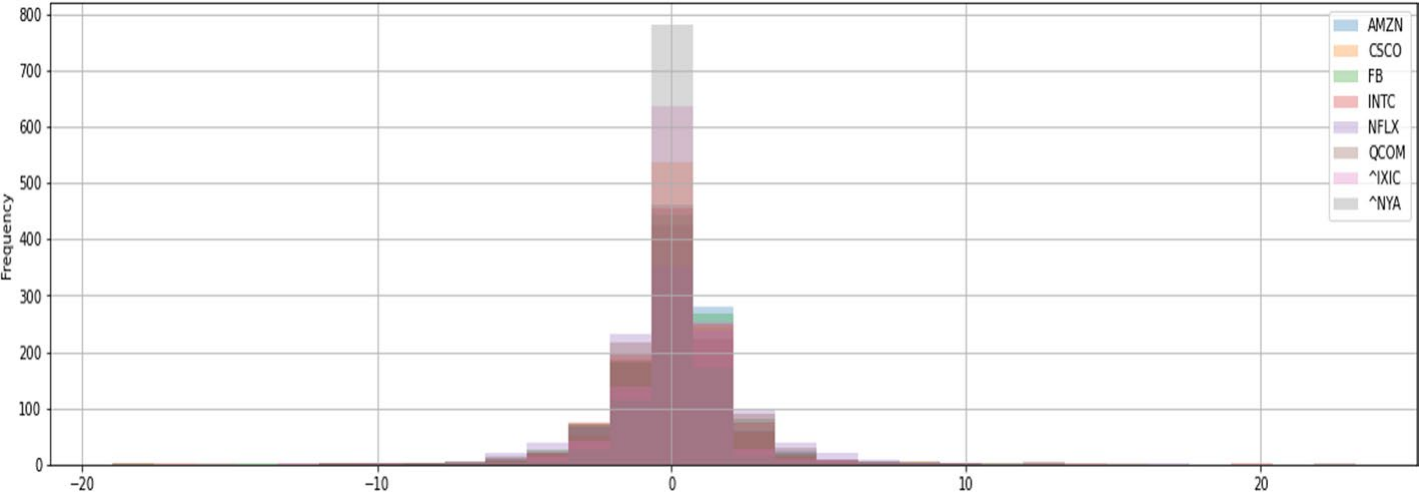
Stock return of sample firms relative to stock market index

Table 2 indicates that the SD of the variables is too large, which may be caused by industries or the pandemic. Therefore, this study takes six additional IoT firms from different industries—including Intel, Netflix, Amazon, Facebook, Cisco, and Qualcomm—and conducts a simple time-series analysis of NASDAQ’s stock prices and NYSE indices plotted in Fig. 1. Figure 1 calculates the underlying stock prices starting from 100 of each firm over the sample time and observes how they changed concerning the stock market index during this period. From the graph’s trend, we can see that the stock prices of Netflix and Amazon, service firms engaged in video streaming and e-commerce, respectively, soared after the COVID-19 outbreak. Conversely, the stock prices of Intel and Qualcomm, two manufacturing firms, performed similarly to the stock market index. These results imply that different industries generate different firm values. Figure 2 shows whether there is volatility clustering and co-movement between the return of the selected firms and the stock market index during the sample period. After the COVID-19 outbreak, the volatility of the six IoT firms and the stock market index became more volatile, with volatility clustering around May 2020. Unlike IoT firms in the service industry, Intel has higher stock return volatility in the manufacturing industry, while Facebook has higher stock return volatility in the service industry. Figure 3 shows the histogram of the stock return distribution of the sample firms and the stock market index, which appears to be a leptokurtic distribution without a fat tail. Careful observation of the coefficient of kurtosis of each stock shows that Facebook is at 2.33, Intel is at 2.78, Cisco is at 2.78, the NYSE is at 1.9, and the NASDAQ is at 2.28. These values are smaller than the typical peak coefficient of 3, indicating a fat-tailed distribution. In other words, using the GARCH model to capture the volatility of spillover effects and volatility clustering is an appropriate choice for subsequent research.

### Correlation analysis

This section presents the correlations between the main research variables. From the correlation matrix in Table 3, it is evident that the excess return of each stock (${R}_{i}-{R}_{f}$) positively correlates with the excess return of the market (${R}_{m}-{R}_{f}$), firm size, innovation spillovers from the AI industry (${\sigma }_{i,AI}^{2}$), innovation spillovers from the fin-tech industry (${\sigma }_{i,Fin-Tech}^{2}$), and total assets and negatively correlated with BMR. If we disregard causality, when a firm has higher systematic risks and a larger scale and receives more innovation spillover effects from industries, it can obtain a higher excess return. Implicitly, the inference of H1 may be correct; however, it is reasonable to discuss the close relationships among these three industries in practice. Conversely, a lower BMR means that the stocks are a growth stock and can reap a higher excess return. The situation is mixed with much noise and solely discusses the relationship between two variables; it must be inferred judiciously with multiple variables. Additionally, similar to Sect. 4.1, this study extracts six IoT firms from different industries to generate a correlation matrix, as shown in Table 4. According to the table, the stock returns of Intel, Netflix, Amazon, Facebook, Cisco, and Qualcomm positively correlate with the NASDAQ and NYSE indices. In other words, they are a group of firms with similar characteristics, and to reduce risks, they should not be in the same portfolio. Furthermore, linear overlap might occur if the least square method is used to estimate the causality of variables; using panel data regression can avoid this dilemma.

**Table 3:** Correlation matrix of the main research variables

	*(*${R}_{i}-{R}_{f}$*)*	*(*${R}_{m}-{R}_{f}$*)*	*BMR*	*SIZE*	${\sigma }_{i,AI}^{2}$	${\sigma }_{i,Fin-Tech}^{2}$	*ASSET*
*(*$${R}_{i}-{R}_{f})$$	1.000000	0.012630	− 0.216441	0.008475	0.001052	0.000862	0.004228
*(*$${R}_{m}-{R}_{f})$$		1.000000	− 0.039362	− 0.007233	− 0.000939	− 0.000770	− 0.002484
BMR			1.000000	− 0.012678	− 0.003910	− 0.003174	− 0.016770
SIZE				1.000000	0.023223	0.012507	− 0.001703
${\sigma }_{i,AI}^{2}$					1.000000	0.746141	− 0.000220
${\sigma }_{i,Fin-Tech}^{2}$						1.000000	− 0.000181
ASSET							1.000000

**Table 4:** Correlation matrix of the stock prices of sample IoT firms

	AMZN	CSCO	FB	INTC	NFLX	QCOM	NASQA	NYSE
AMZN	1.000000	0.486586	0.631652	0.466687	0.634825	0.436964	0.753804	0.465596
CSCO	0.486586	1.000000	0.447883	0.581133	0.384141	0.458985	0.720474	0.709188
FB	0.631652	0.447883	1.000000	0.451196	0.502508	0.407571	0.726555	0.528547
INTC	0.466687	0.581133	0.451196	1.000000	0.412319	0.521336	0.701465	0.626347
NFLX	0.634825	0.384141	0.502508	0.412319	1.000000	0.376621	0.610958	0.382842
QCOM	0.436964	0.458985	0.407571	0.521336	0.376621	1.000000	0.652960	0.562331
DASQA	0.753804	0.720474	0.726555	0.701465	0.610958	0.652960	1.000000	0.853634
NYSE	0.465596	0.709188	0.528547	0.626347	0.382842	0.562331	0.853634	1.000000

**Fig. 4:**
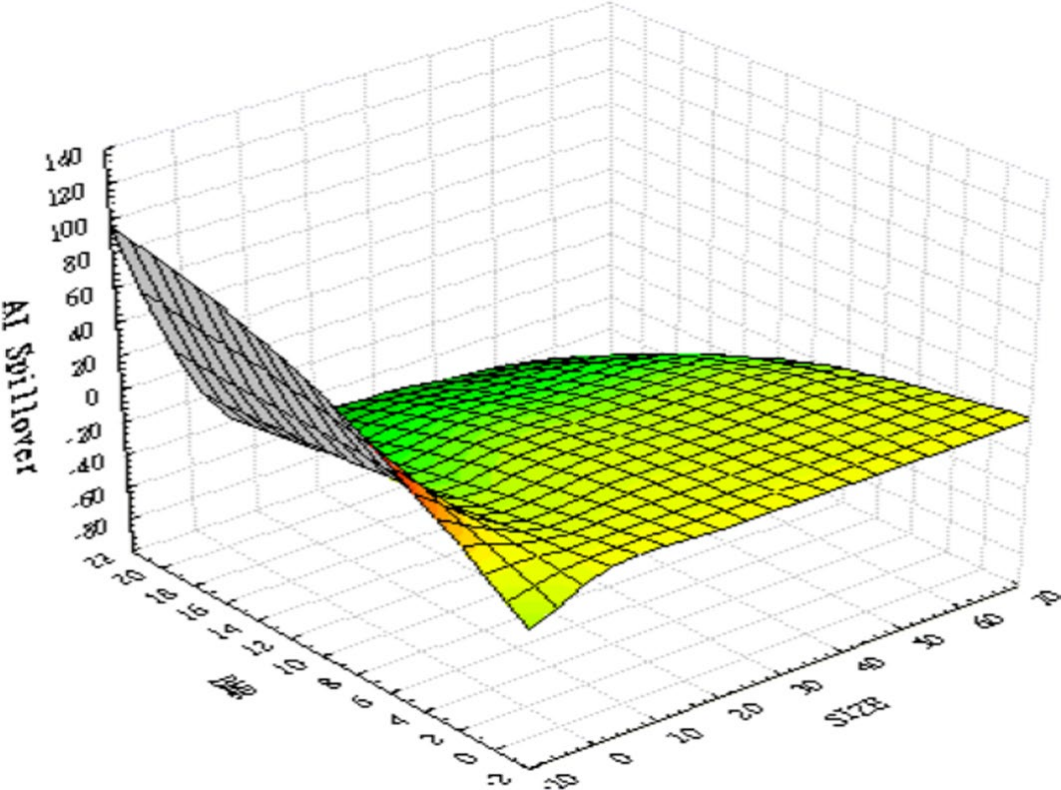
Standardized numerical surface plot for (${R}_{i}-{R}_{f}$), (${\sigma }_{i,AI}^{2}$), and (${\sigma }_{i,Fin-Tech}^{2}$)

**Fig. 5:**
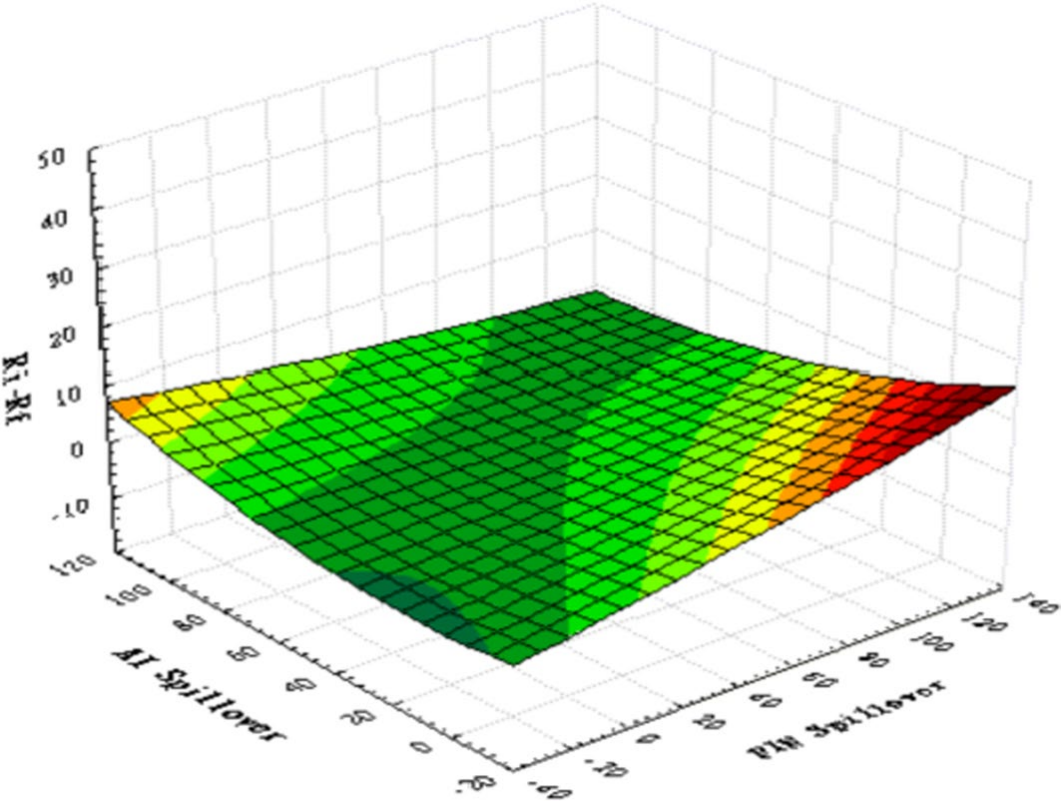
Standardized numerical surface plot for firm size, BMR, and (${\sigma }_{i,AI}^{2}$)

**Fig. 6:**
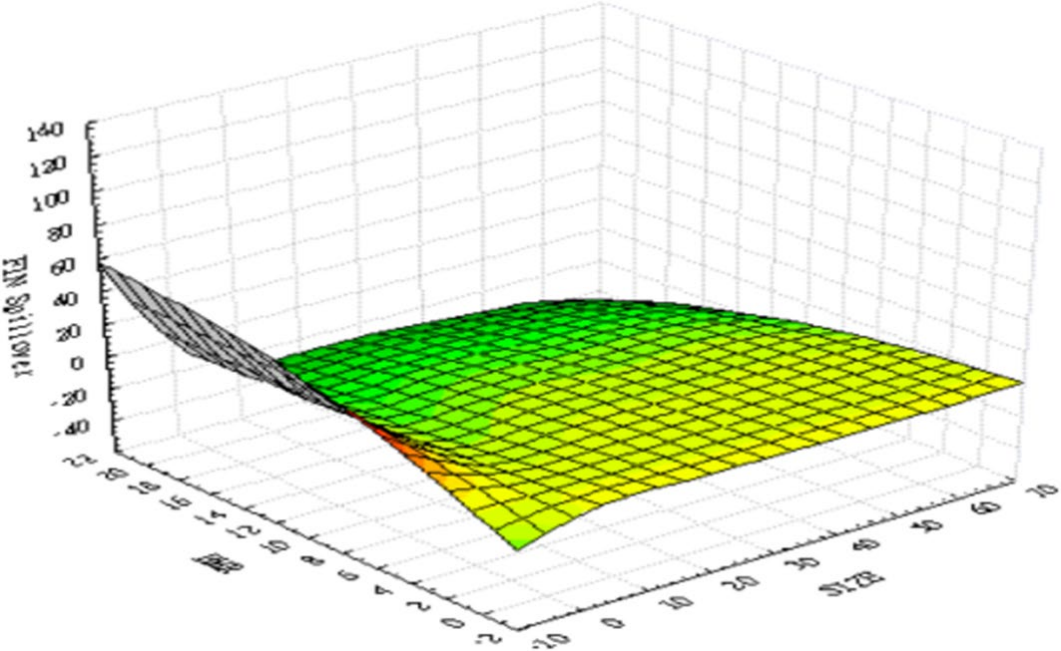
Standardized numerical surface plot for firm size, BMR, and (${\sigma }_{i,Fin-Tech}^{2}$)

**Fig. 7:**
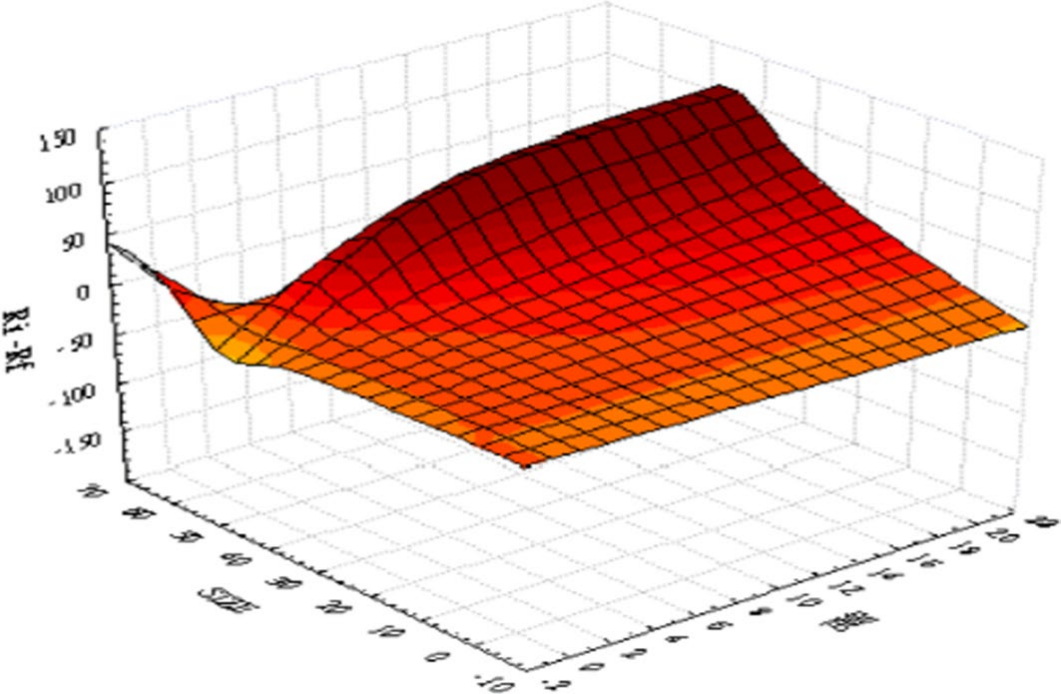
Standardized numerical surface plot for (${R}_{i}-{R}_{f}$), firm size, and BMR

**Fig. 8:**
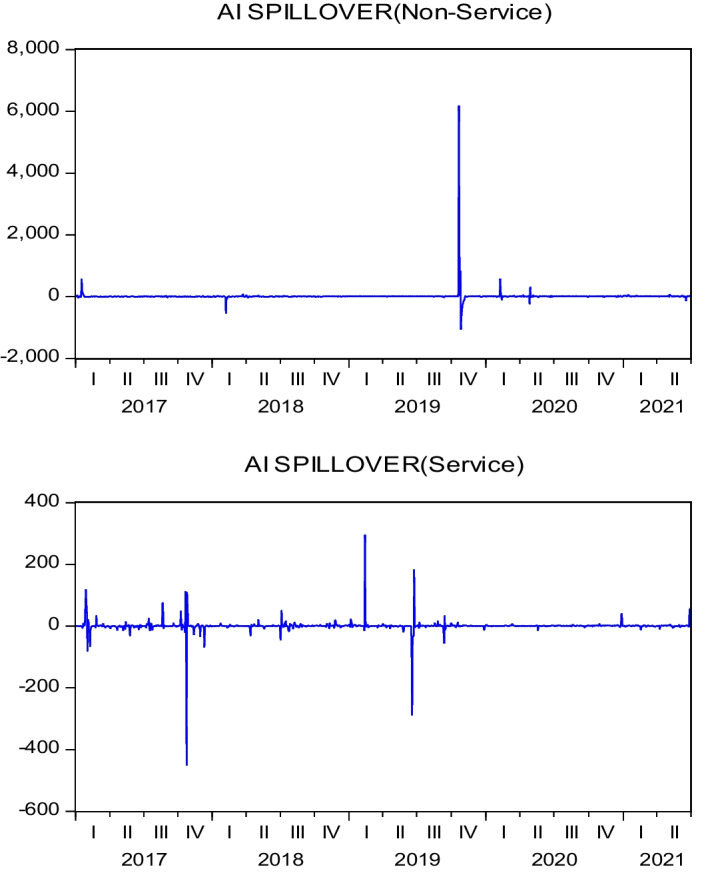
Trends of Spillover Effects from the AI Industry

**Fig. 9:**
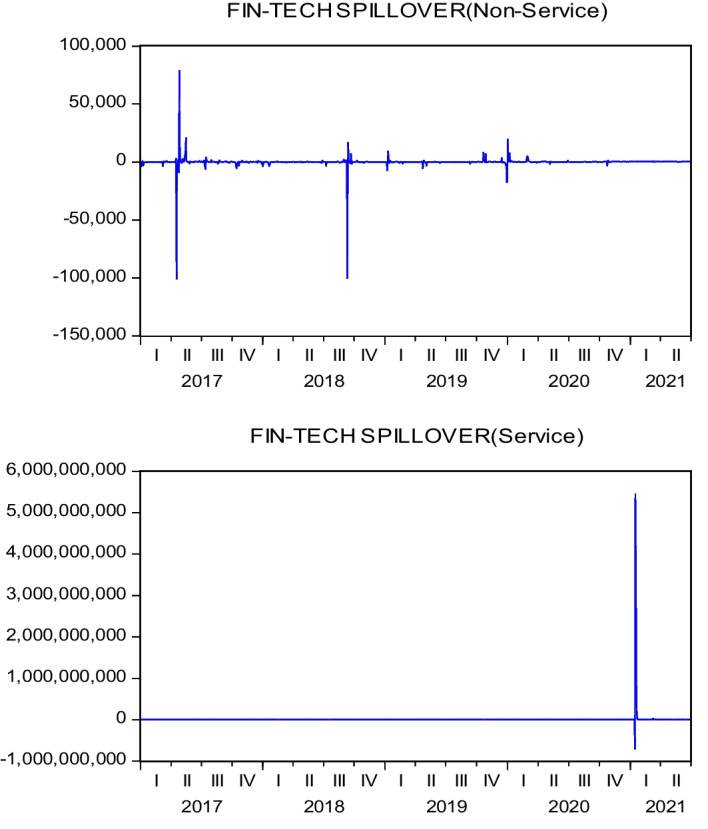
Trends of Spillover Effects from the Fin-tech Industry

As the difference in values between the variables is too large for graphical illustration, we first standardize all the data before plotting 3D graphics to express the IoT firms’ characteristic trends. Figure 4 presents the relationships between the three variables—namely, (${R}_{i}-{R}_{f}$), (${\sigma }_{i,AI}^{2}$), and (${\sigma }_{i,Fin-Tech}^{2}$). The surface plot trend reveals that the higher the value of (${\sigma }_{i,AI}^{2}$) or (${\sigma }_{i,Fin-Tech}^{2}$), the higher the excess return of each stock (${R}_{i}-{R}_{f}$). This tentatively confirms a homogeneous relationship between excess return and innovation spillover effects; thus, H1 may be supported. Figure 5 plots the relationships between size, BMR, and (${\sigma }_{i,AI}^{2}$). The surface plot shows that smaller-value stocks are more affected by the innovation spillovers (${\sigma }_{i,AI}^{2}$) from the AI industry. Compared with Fig. 6, the relationships among firm size, BMR, and (${\sigma }_{i,Fin-Tech}^{2}$) reveal that smaller-value stocks are more affected by the innovation spillovers from the fin-tech industry (${\sigma }_{i,Fin-Tech}^{2}$). Next, this study plots Fig. 7 to show the relationships between (${\sigma }_{i,Fin-Tech}^{2}$), firm size, and BMR. The plot results show that larger-value stocks can obtain higher excess returns (${R}_{i}-{R}_{f}$). In other words, to invest in an IoT firm in the U.S. market to obtain a better stock return, one can choose larger-value stocks that are more affected by the innovation spillovers of industries. Finally, this paper plots the time-series trends of the spillover effects of the two industries on IoT, as shown in Figs. 8 and 9. In summary, volatility spillover effects from the service industry IoT are more frequent in the AI industry, while the volatility spillover effects from the non-service industry IoT are more sensitive to the fin-tech industry.

### Panel data regression

According to the analysis results in Sects. 4.1 and 4.2, there may be fat-tailed distributions of the main research variables. Nevertheless, there are problems with volatility clustering and linear overlap. More importantly, some hidden factors in time have not yet been observed, possibly environmental changes or industrial attributes, which can affect the excess return of stocks. Thus, this study uses panel data regression for analysis. Before running the model, we stack the data of sample firms and then establish a more robust estimation method considering the random variation of residual (${\varepsilon }_{i,t}$), that is, ${\varepsilon }_{i,t}$=${\mu }_{i}+{\lambda }_{i}+{\nu }_{i,t}$, where ${\mu }_{i}$ and ${\nu }_{i,t}$ denote random variation, and ${\lambda }_{i}$ is the time effect. This study analyzes the five models in Table 5; the approach is first to choose period random effects for estimation and then conduct the Hausman test to determine whether the null hypothesis (H_0_: random effect) is valid. From the five models’ test reports, all the *p*-values of the Hausman statistics are high. H_0_ is not rejected; thus, using period random effects for estimation is appropriate.

**Table 5:** Panel data regression

*Depend Variable*	Model 1		Model 2		Model 3		Model 4		Model 5	
$({R}_{i,t}-{R}_{f,t})$	Coefficient	t-statistic	Coefficient	t-statistic	Coefficient	t-statistic	Coefficient	t-statistic	Coefficient	t-statistic
Intercept	− 0.0199***	− 2.88789	− 0.72976***	− 37.80125	− 0.02475***	− 12.04331	− 0.00250***	− 3.723995	− 0.06670***	− 3.31868
(${R}_{m}-{R}_{f}$)	1.24E − 05***	11.9587	0.00011***	36.15421	3.52E − 06	1.636524	0.86485***	35.46794	4.70E − 06	0.97005
BMR	− 0.1124***	− 18.8808	− 0.10402***	− 18.61919	− 0.00907***	− 12.34377	− 7.80E − 05	− 0.684454	− 0.02787***	− 8.59256
Size	1.27E − 07***	7.785249	9.27E − 08***	7.619385	2.98E − 08	0.322771	5.65E − 09	0.466804	0.00340*	1.66917
D			− 0.34246***	− 20.55658	0.02549***	8.404843	0.00124	1.615604	0.07113**	2.18626
H			1.03161***	34.14187						
${\sigma }_{i,AI,t-1}^{2}$	4.86E − 13***	3.431620	1.41E − 12***	3.222290	1.02E − 08**	2.238173	− 1.14E − 07***	− 3.209640	2.29E − 07***	5.46373
${\sigma }_{i,Fin-Tech,t-1}^{2}$	1.95E − 13***	3.151131	**7.78E − 14**	0.547852	− 2.84E − 07**	− 2.237765	6.74E − 08***	3.933989	− 6.37E − 06***	− 5.46381
D*(${R}_{m}-{R}_{f}$)					0.99974***	1321.892	0.01446	0.497166	0.99973***	427.445
D*BMR					0.00877***	10.16661	− 0.00017	− 1.371775	0.02724***	5.40659
D*Size					− 1.61E − 08	− 0.171484	8.71E − 09	0.709120	− 0.00356	− 1.09197
D*$({\sigma }_{i,AI,t-1}^{2}$)					− 1.02E − 08**	− 2.238171	1.14E − 07***	3.209641	**1.76E − 06**	0.57349
D*(${\sigma }_{i,Fin-Tech,t-1}^{2}$)					2.84E − 07**	2.237767	− 6.74E − 08***	− 3.933981	6.95E − 06***	2.84766
*N*	58,589		58,589		58,589		46,690		11,899	
Adj. R^2^	0.046807		0.095833		0.969676		0.101251		0.96930	
F − statistic	576.3947***		888.1092***		170318.1***		481.1156***		34156.09***	

Major model ${R}_{i,t}-{R}_{f,t}={{\alpha }_{0s(ns)\_pre-covid}+(\alpha }_{0s(ns)\_covid}-{\alpha }_{0s(ns)\_pre-covid})D+{\beta }_{1s(ns)\_pre-covid}{(R}_{m,t}-{R}_{f,t})+({\beta }_{1s(ns)\_covid}-{\beta }_{1s(ns)\_pre-covid})D{(R}_{m,t}-{R}_{f,t})+{\beta }_{2s(ns)\_pre-covid}ln{\left(SIZE\right)}_{i,t}+({\beta }_{2s(ns)\_covidS}-{\beta }_{2s(ns)\_pre-covid})Dln{\left(SIZE\right)}_{i,t}+{\beta }_{3s(ns)\_pre-covid}{(BMR)}_{i,t}+({\beta }_{3s(ns)\_covid}-{\beta }_{3s(ns)\_pre-covid}){D(BMR)}_{i,t}+{\beta }_{4s(ns)\_pre-covid}{({\sigma }_{i,AI}^{2})}_{t-1}+({\beta }_{4s(ns)\_covid}-{\beta }_{4s(ns)\_pre-covid})D{({\sigma }_{i,AI}^{2})}_{t-1}+{\beta }_{5s(ns)\_pre-covid}{({\sigma }_{i,Fin-Tech}^{2})}_{t-1}+({\beta }_{5s(ns)\_covid}-{\beta }_{5s(ns)\_pre-covid})D{({\sigma }_{i,Fin-Tech}^{2})}_{t-1}+{\varepsilon }_{i,t}$, ${\varepsilon }_{i,t}$=${\mu }_{i}+{\lambda }_{i}+{\nu }_{i,t}$; Model 1 examines H1, Model 3 examines the full sample, Model 4 examines H2, and Model 5 examines H3; each model lists the coefficients and t-values of period random effects; *** (*P* < 0.01) and ** (*P* < 0.05) are statistically significant; the significance of main variables is shown in bold

The five models in Table 5 describe different scenarios. Model 1 comprises the fundamental three factors of Fama–French (1996) and the independent variables of innovation spillovers from the two industries. As shown by the coefficients of Model 1, IoT firms’ excess returns are significantly influenced by all the independent variables, including firm size, systematic risk (${R}_{m}-{R}_{f}$), and the innovation spillovers from the AI industry (${\sigma }_{i,AI,t-1}^{2}$), and they are significantly and positively correlated with the innovation spillovers from the fin-tech industry (${\sigma }_{i,Fin-Tech,t-1}^{2}$); however, they are significantly and negatively correlated with intercept and BMR. Model 1’s findings are in the same direction as the aforementioned single-factor descriptive statistics, where there are also significant positive correlations with the variables ${\sigma }_{i,AI,t-1}^{2}$(4.86E-13***, *p* < 0.01) and ${\sigma }_{i,Fin-Tech,t-1}^{2}$(1.95E-13***, *p* < 0.01). In other words, IoT firms are strongly receptive to the innovation spillovers from related industries during this period and absorb their knowledge to build profitability. The findings echo Rogers (1995, 2002), Dietzenbacher (2000), and Fleisch (2010) and support H1 in this study, implying that the higher the acceptance level of the innovation spillovers from the AI and fin-tech industries, the better the performance of the IoT firms.

Model 2 adds the dummy variables of environmental changes and industries to understand whether they affect the excess return of IoT stocks and the innovation spillovers from the AI (${\sigma }_{i,AI,t-1}^{2}$) and the fin-tech (${\sigma }_{i,Fin-Tech,t-1}^{2}$) industries. The coefficients of Model 2 indicate that the excess return of IoT firms is significantly affected by most of the independent variables—significantly positively correlated to firm size, systematic risk (${\sigma }_{i,Fin-Tech,t-1}^{2}$), industry effect (H), and ${\sigma }_{i,AI,t-1}^{2}$; however, they are significantly and negatively correlated with intercept, environmental change (D), and BMR. Remarkably, although the excess return of stocks (${R}_{i}-{R}_{f}$) is positively correlated with ${\sigma }_{i,Fin-Tech,t-1}^{2}$, it is not significant. From the Model 2 results, it is evident that the COVID-19 pandemic affects IoT firms’ excess returns—that is, it causes structural changes. The findings are similar to Singha et al. (2020); industrial classification also has explanatory power for the excess return of stocks (${R}_{i}-{R}_{f}$). After loading the data into the model, the explanatory power of ${\sigma }_{i,Fin-Tech,t-1}^{2}$ on (${R}_{i}-{R}_{f}$) disappears, implying that before and after the COVID-19 outbreak, different industries handled innovation spillovers from the fin-tech industry in different directions and offset each other to make the explanatory power insignificant.

Model 2 shows that environmental changes and different industries may change the dependence between the main research variables. Therefore, it is necessary to observe the influence of environmental changes on the excess return of stocks (${R}_{i}-{R}_{f}$) in the two intervals before and after the COVID-19 outbreak. Model 3 organizes the coefficients, indicating that before the pandemic, the acceptance levels of the innovation spillovers from the AI industry (${\sigma }_{i,AI,t-1}^{2}$) of all the IoT firms were significantly and positively correlated with the excess return of stocks (1.02E-08**, *P* < 0.05); however, the innovation spillovers’ acceptance levels from the fin-tech industry (${\sigma }_{i,Fin-Tech,t-1}^{2}$) were significantly negatively correlated (− 2.84E-07**, *P* < 0.05). Conversely, after the COVID-19 outbreak, the innovation spillovers’ acceptance levels from the AI industry (D ∗ ${\sigma }_{i,AI,t-1}^{2}$) of the IoT firms gradually decreased (− 1.02E-08**, *P* < 0.05), while the innovation spillovers’ acceptance levels from the fin-tech industry (D*${\sigma }_{i,Fin-Tech,t-1}^{2}$) gradually increased (2.84E-07**, *P* < 0.05). Overall, after the COVID-19 outbreak, the IoT firms underwent changes and corporate transformation. Some firms focused on AI transformation and application, while others invested in fin-tech technologies. It is still unknown how firms’ industrial attributes influence innovation spillovers from the two related industries; however, it is inevitable that IoT firms adjust their innovation spillovers’ acceptance levels from industries based on their interests (Globerman 1979; Blomstrom and Persson 1983; Dietzenbacher 2000; Lu et al. 2021) and develop strategies to respond to market demands (Fleisch 2010). Remarkably, firm size and systematic risk (${R}_{m}-{R}_{f}$) in the Fama–French (1996) model became insignificant because of the COVID-19 pandemic, implying that traditional asset pricing may be subject to significant forecasting bias under major environmental changes.

According to Model 3, IoT firms were more receptive to the spillover effects from the fin-tech industry before and after the COVID-19 outbreak; however, the AI industry showed the opposite result, possibly due to the firm’s industrial category. As Hypotheses 2 and 3 suggest, during the pandemic, different strategies of service and non-service industries may have been developed based on interests and resource constraints. Therefore, we further differentiate the data into Models 4 and 5 for analysis. Model 4 is the data of the service industry, showing a gradual increase (1.14E-07***, *P* < 0.01) in the innovation spillovers’ acceptance levels from the AI industry (D*${\sigma }_{i,AI,t-1}^{2}$), with a gradual decrease (− 6.74E-08***, *P* < 0.01) from the fin-tech industry (D*${\sigma }_{i,Fin-Tech,t-1}^{2}$). Model 5 is the data of the non-service industry, showing a gradual increase in innovation spillovers from both the AI and fin-tech industries; however, the direction for the AI industry (D*${\sigma }_{i,AI,t-1}^{2}$) is consistent with the inference but not significant. The results echo the research of Guo (2018) and Aysun and Yom (2021). The data in Model 5 occurs because capital investment and regional clustering essentially take time and are not instantaneous. Such results also confirm that Model 3’s findings are mixed with the industry effect and that not all IoT firms were equally receptive or oriented to the spillover effects from specific industries before and after the COVID-19 outbreak. In principle, Models 4 and 5 support H2 and H3, while the three-factors Fama–French (1996) exhibit different results for systematic risk (${R}_{m}-{R}_{f}$) and BMR in different models. Table 5 presents the coefficients of other variables. In summary, Model 3 has the largest adjusted r-squared (Adj. R^2^) value, indicating that the environmental variable information is essential for the pricing of capital assets; all models have significant F-values, indicating that it was a good choice to add the factor of innovation spillovers from industries to forecast the excess return of IoT firms.

In summary, Model 3 shows all the aggregated effects of IoT companies’ acceptance of innovation spillovers of AI and fin-tech industries before and after the COVID-19 outbreak. Compared to the Model 1 and 2 results, once the environmental changes factor was included, Model 3’s overall adj R^2^ rose significantly. In other words, internal transformations or organizational reforms brought by environmental changes did affect enterprise value. Additionally, this phenomenon varies in different industries. Model 4 demonstrates the aggregated effect of the acceptance of innovation spillovers of relevant industries by IoT companies in the service industry before and after the COVID-19 outbreak. Environmental changes brought about internal reforms to enterprises and changed their value; however, they were not as crucial to the overall model because the model’s increase in adj R^2^ was not high. In contrast, after the same model was applied to IoT companies outside the service industry, Model 5’s adj R^2^ surged. This surge implies that such companies were affected by environmental changes, meaning their value was sensitive to internal transformations and reforms. Environmental changes were a significant factor in the model assessment. Fama–French (1996)’s model revealed that some companies’ values were affected by three factors. This study suggests that organizational reforms arising from environmental changes can affect companies’ performance and the importance of environmental changes can vary by industry.

We next investigate why enterprise performances are affected by their global transformations or organizational reforms in the event of an impact of innovation spillovers. The four variable coefficients, D*BMR, D*Size, D*(${\sigma }_{i,AI,t-1}^{2}$), and D*(${\sigma }_{i,Fin-Tech,t-1}^{2}$), in Models 4 and 5 indicate that enterprises respond to environmental changes. Cassiman and Veugelers (2002) and Cohen and Levinthal (1989) indicated that companies’ performance benefits from the innovation spillovers of industries and requires R&D input and innovative technologies, which are critical to future competition. According to the data comparison between Models 4 and 5, IoT companies outside the service industry enjoyed the benefit of industry clusters to take advantage of a favorable geographical position to conduct R&D cooperation and technological transfer or authorization, improving their profitability and market shares. Therefore, environmental changes made a higher contribution to Model 5. Comparatively, IoT companies in the service industry followed their business models, meaning that their strengths lay outside R&D and technology. Though their performance reflected innovation spillovers, such companies only took advantage of sudden changes to make short-term investments. Hence, adj R^2^ in Model 4 changed slightly.

### Robustness analysis

This study included the innovation spillovers from industries in the model of Fama–French (1996) for discussion. Table 5 presents the preliminary conclusions. Because different proxy variables or the addition of independent variables may affect the study results, it is necessary to conduct a robustness analysis. Models 6, 7, and 8 in Table 6 are a continuation of the panel data regression; the proxy variable of firm size in the model of Fama–French (1996) is replaced based on the number of outstanding shares to total assets (Ln [ASSET]), and the random effects for model estimation are estimated first. According to the estimation reports, the *p*-values of the Hausman statistics do not reject H0; thus, it is appropriate to use period random effects. From the coefficients in Model 6, it is evident that the excess returns of IoT firms significantly and positively correlate with all the independent variables—that is, firm size, systematic risk (${R}_{m}-{R}_{f}$), innovation spillovers from the AI industry (${\sigma }_{i,AI,t-1}^{2}$), and innovation spillovers from the fin-tech industry (${\sigma }_{i,Fin-Tech,t-1}^{2}$); however, they are significantly negatively correlated with intercept and BMR. All the signs and directions are the same as Model 1’s conclusions; thus, H1 is supported. Model 7 examines the data of the service IoT firms. The innovation spillovers’ acceptance levels from the AI industry (D ∗ ${\sigma }_{i,AI,t-1}^{2}$) of the IoT firms gradually increased (1.15E-07***, *P* < 0.01), while those from the fin-tech industry ($\mathrm{D}*{\sigma }_{i,Fin-Tech,t-1}^{2}$) gradually decreased (− 6.51E-08***, *P* < 0.01). The results are the same as those of Model 4; thus, H2 is supported. In contrast, Model 8 examines non-service IoT firms. The data shows gradual increases in the innovation spillovers’ acceptance levels from both the AI (D ∗ ${\sigma }_{i,AI,t-1}^{2}$) and fin-tech ($\mathrm{D}*{\sigma }_{i,Fin-Tech,t-1}^{2}$) industries. Although the coefficients are insignificant in the fin-tech industry (D ∗ ${\sigma }_{i,AI,t-1}^{2}$), the directions are the same. Thus, H3 is supported.

**Table 6:** Robustness analysis

*Depend Variable*	Model 6		Model 7		Model 8		Model 9		Model 10	
$({R}_{i,t}-{R}_{f,t})$	Coefficient	t-statistic	Coefficient	t-statistic	Coefficient	t-statistic	Coefficient	t-statistic	Coefficient	t-statistic
Intercept	− 0.01913***	− 2.767963	− 0.00820***	− 8.367746	− 0.07094***	− 7.366044	− 0.00250***	− 3.723975	− 0.070238	− 1.172472
(${R}_{m}-{R}_{f}$)	1.22E − 05***	11.86222	0.85474***	35.03743	1.32E − 06	0.265783	0.86485***	35.46774	1.96E − 06	0.523152
BMR	− 0.11250***	− 18.88183	0.00017	1.500401	− 0.03253***	− 10.60821	− 7.80E − 05	− 0.684450	− 0.032641	− 0.880640
Asset	3.09E − 06***	3.659897	0.00065***	7.993936	− 4.48E − 06	− 0.532548				
Size							5.65E − 09	0.466802	− 0.000282	− 0.066990
D			0.00763***	5.713188	0.18520***	4.113677	0.00124	1.621585	0.105553	1.076105
${\sigma }_{i,AI,t-1}^{2}$	8.90E − 13***	4.252773	− 1.15E − 07***	− 3.239342	5.08834***	2.238173	− 1.14E − 07***	− 3.209619	2.13E − 07	0.816159
${\sigma }_{i,Fin-Tech,t-1}^{2}$	1.38E − 13**	2.097997	6.51E − 08***	3.796569	− 5.08850***	− 2.237765	6.74E − 08***	3.933979	− 5.92E − 06	− 0.816184
D*(${R}_{m}-{R}_{f}$)			0.026691	0.916722	1.00311**	380.3264	0.01470	0.505392	1.002839	440.6452
D*BMR			− 0.000456***	− 3.358283	0.02790**	5.500631	− 0.00017	− 1.372978	0.031740	0.885858
D*Asset			− 0.000701***	− 5.542301	− 0.00191	− 0.470347	8.75E − 09	0.712404	0.007333	0.762140
D*$({\sigma }_{i,AI,t-1}^{2}$)			1.15E − 07***	3.239344	**1.49E − 06**	0.459562	1.14E − 07***	3.209628	1.34E − 06**	2.227196
D*(${\sigma }_{i,Fin-Tech,t-1}^{2}$)			− 6.51E − 08***	− 3.796561	6.50E − 06**	2.526280	− 6.74E − 08***	− 3.933967	**6.82E − 06**	0.908963
${\sigma }_{i,AI,t}^{2}$							− 5.38E − 13	− 0.683252	1.52E − 07	0.778654
${\sigma }_{i,Fin-Tech}^{2}$							− 3.05E − 14	− 0.065451	− 4.22E − 06	− 0.778672
*N*	58,589		46,690		11,899		46,690		11,899	
Adj. R	0.046774		0.101584		0.969655		0.101229		0.969712	
F − statistic	575.9601***		482.8721***		322.6276***		407.1542***		322.7338***	

Robustness models: ${R}_{i,t}-{R}_{f,t}={{\alpha }_{0s{\left(ns\right)}_{pre}-covid}+(\alpha }_{0s{\left(ns\right)}_{covid}}-{\alpha }_{0s{\left(ns\right)}_{pre}-covid})D+{\beta }_{1s{\left(ns\right)}_{pre}-covid}{(R}_{m,t}-{R}_{f,t})+({\beta }_{1s{\left(ns\right)}_{covid}}-{\beta }_{1s{\left(ns\right)}_{pre}-covid})D{(R}_{m,t}-{R}_{f,t})+{\beta }_{2s{\left(ns\right)}_{pre}-covid}ln{\left(ASSET, SIZE\right)}_{i,t}+({\beta }_{2s{\left(ns\right)}_{covidS}}-{\beta }_{2s{\left(ns\right)}_{pre}-covid})Dln{\left(SIZE\right)}_{i,t}+{\beta }_{3s{\left(ns\right)}_{pre}-covid}{\left(BMR\right)}_{i,t}+({\beta }_{3s{\left(ns\right)}_{covid}}-{\beta }_{3s{\left(ns\right)}_{pre}-covid}){D\left(BMR\right)}_{i,t}+{\beta }_{4s{\left(ns\right)}_{pre}-covid}{\left({\sigma }_{i,AI}^{2}\right)}_{t-1}+({\beta }_{4s{\left(ns\right)}_{covid}}-{\beta }_{4s{\left(ns\right)}_{pre}-covid})D{\left({\sigma }_{i,AI}^{2}\right)}_{t-1}+{\beta }_{5s{\left(ns\right)}_{pre}-covid}{\left({\sigma }_{i,Fin-Tech}^{2}\right)}_{t-1}+({\beta }_{5s{\left(ns\right)}_{covid}}-{\beta }_{5s{\left(ns\right)}_{pre}-covid})D{\left({\sigma }_{i,Fin-Tech}^{2}\right)}_{t-1}+{\beta }_{6s(ns)}\left({\sigma }_{i,AI,t}^{2}\right)+{\beta }_{7s(ns)}\left({\sigma }_{i,Fin-Tech,t}^{2}\right)+{\varepsilon }_{i,t}$, ${\varepsilon }_{i,t}$=${\mu }_{i}+{\lambda }_{i}+{\nu }_{i,t}$ (*p* < 0.01); Model 6 examines H1, Model 7 and 9 examine H2, and Model 8 and 10 examine H3; each model lists the coefficients and t-values of period random effects; *** (*P* < 0.01), and ** (*P* < 0.05) are statistically significant; the significance of the main variables is shown in bold

Regarding Models 9 and 10, two current independent variables—the innovation spillovers from both the AI (${\sigma }_{i,AI}^{2}$) and fin-tech (${\sigma }_{i,Fin-Tech}^{2}$) industries—are added to the original model to test whether H2 and H3 remain valid. Similarly, we first choose period random effects for model estimation. The results show that the *p*-values of the Hausman statistics do not reject H0; thus, using period random effects is appropriate. The data from Model 9 show that the innovation spillovers’ acceptance levels from the AI industry (D ∗ ${\sigma }_{i,AI,t-1}^{2}$) of service firms gradually increased (1.14E-07***, *P* < 0.01) while the fin-tech industry ($\mathrm{D}*{\sigma }_{i,Fin-Tech,t-1}^{2}$) gradually decreased (− 6.74E-08***, *P* < 0.01). The results are the same as those of Models 4 and 7; thus, H2 is supported. According to Model 10’s coefficients, the innovation spillovers’ acceptance levels from both the AI (D ∗ ${\sigma }_{i,AI,t-1}^{2}$) and fin-tech ($\mathrm{D}*{\sigma }_{i,Fin-Tech,t-1}^{2}$) industries of non-service firms gradually increased after the coronavirus outbreak. Although the coefficients for the fin-tech industry ($\mathrm{D}*{\sigma }_{i,Fin-Tech,t-1}^{2}$) are insignificant, the direction is the same; thus, H3 is supported. Tables 5 and 6 show no change in direction, indicating that the inferences in H1, H2, and H3 exist and that the results are robust.

The robustness analysis supports the three hypotheses; IoT firms are affected by cross-industry innovation spillovers during the study period even when the proxy variables are replaced, and the current effects are increased. The findings echo Dietzenbacher (2000) and Diebold and Yilmaz (2012); however, the relationship between excess return of stock prices and spillover effects also increases or decreases across industries, environments, and leader–laggard scenarios. This is related to the gradual-information-diffusion hypothesis proposed by Hong et al. (2007) and the claims of Rehman and Narayan (2021) that financial markets are subject to changes in policy and environment.

## Conclusions and recommendations

This study aims to determine whether the innovation spillovers in relevant industries can change the value of specific companies and whether the value is affected by companies’ business characteristics or environmental changes. We use Python to download quality public information from Yahoo Finance, a cost-effective approach, and then apply the GARCH model to capture the influence of innovation spillovers from industries on stocks and obtain the variables independent of the factor zoo. The empirical findings support the three hypotheses and the claims of Dietzenbacher (2000) and Diebold and Yilmaz (2012) that cross-industry innovation diffusion can contribute to domestic economic growth and productivity improvement. Moreover, Jaffe (1986) and Bernstein and Nadiri (1989) argued that productivity improvement contributes to cost reductions and competitiveness and that stock prices reflect the result. Chen et al. (2013) also indicated that firms continuously affected by innovation spillovers have abnormal payoffs in the long run, which is consistent with this study’s regression findings. Furthermore, Qarni and Gulzar (2021) found asymmetric volatility across foreign exchange markets, while Kang et al. (2017) and Rehman and Narayan (2021) showed that international oil prices are affected by economic policy uncertainty and sentiment proxies and that financial markets have a two-way spillover relationship with the oil industry. As Hong et al. (2007) suggested in the gradual-information-diffusion hypothesis, policies that appear unrelated to an industry can eventually affect another market through the transmission to indices but with a leader–laggard relationship. In this paper’s robustness analysis, we also see that the lagging term of innovation spillovers significantly influences stock prices, which indirectly supports the gradual-information-diffusion hypothesis.

This paper’s main contribution is to use multi-year data to observe the impact of the transmission mechanism of internal and external variables on the change in the value of IoT firms. More innovation spillovers from the relevant industry do not necessarily improve the company’s share price. It also has to do with the environment, the company’s characteristics, and the organizational style, particularly the firm’s response to the arrival of spillover, which has rarely been discussed in the past literature. While this study assumes that firms absorb innovation spillovers according to their capacity, Cohen and Levinthal (1989) believed that it would not be complete unless R&D is used as a pointer to absorptive capacity. Thus, this variable could be added as a common explanatory factor in future models. In addition, Diebold and Yilmaz (2012) and Kang et al. (2017) used VAR as the research model; thus, future models could add explanatory factors to the established model or explore the two-way spillover relationship between two related industries and financial markets to compare the differences in findings. Last, we can implement policy leverage to accelerate the upgrading and transformation of innovation-interactive industries by following the practices of Singapore and South Korea, which invest in or assist in developing specific industries with sovereign wealth funds and providing tax incentives. It is undoubtedly essential to control risk spillovers from related industries.

## Data Availability

We searched on the webpage of Yahoo Finance and used two key instructions—namely, Internet content and information—and information technology services to screen and deduct the duplicate firms, obtaining 50 firms that met the definition. Then, we ran the Python program to download the firms’ stock prices and financial statements announced on Yahoo Finance. The financial statements, financial ratios, market capitalization, and stock liquidity data provided by Yahoo Finance were sourced from Morningstar; and the historical data and daily updates of U.S. stocks and global indices were sourced from Commodity Systems, Inc. In other words, the data quality is rigorous in this study.
